# Supplementary material for: HIBRID: histology-based risk-stratification with deep learning and ctDNA in colorectal cancer
Source: Nat Commun. 2025 Aug 14;16:7561. doi: 10.1038/s41467-025-62910-8 (PMC12354865; doi:10.1038/s41467-025-62910-8)
Supplement: Supplementary file 1 — Supplementary Information [file 41467_2025_62910_MOESM1_ESM.pdf]

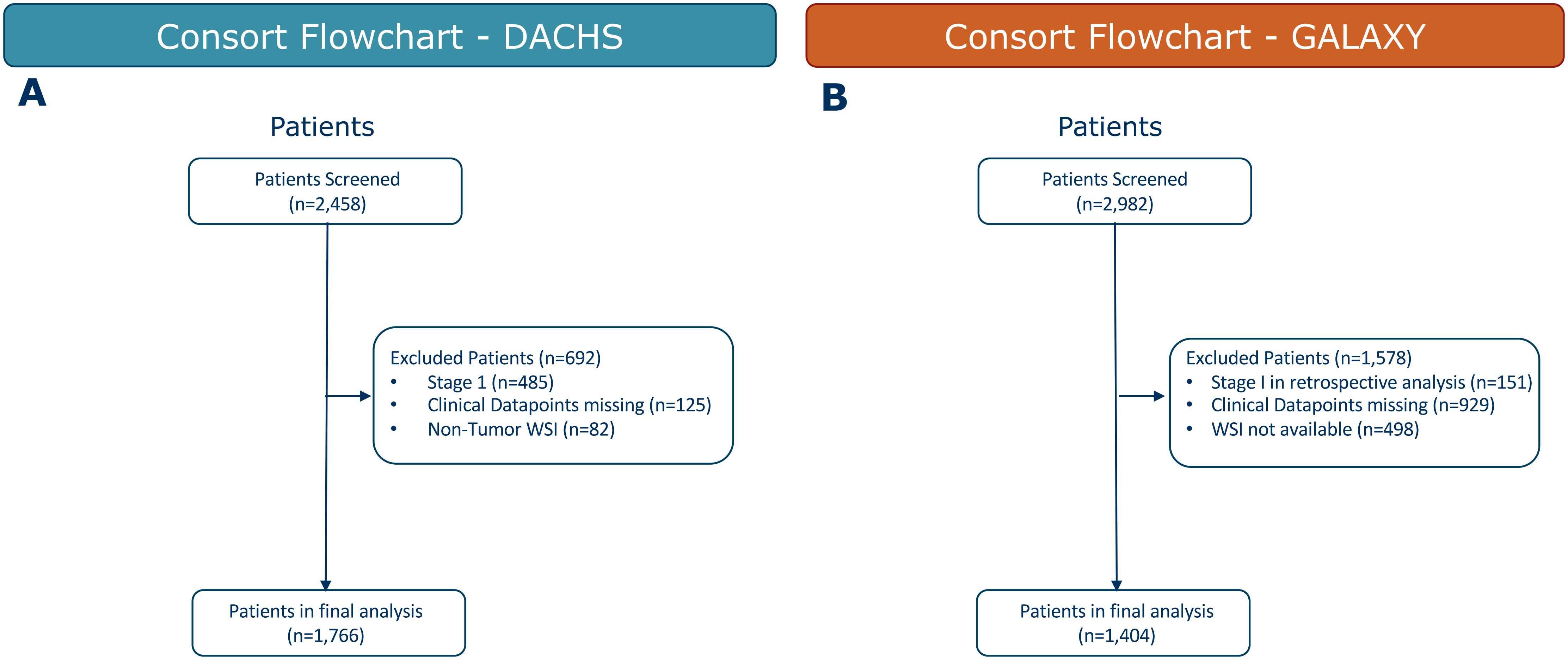

Supplementary Figure 1: Consort diagram for both cohorts

Flowchart showing initial screened patients and exclusion criteria's for (A) the DACHS cohort and (B) the GALAXY cohort. DACHS=Darmkrebs: Chancen der Verhütung durch Screening Study, WSI= Whole Slide Image

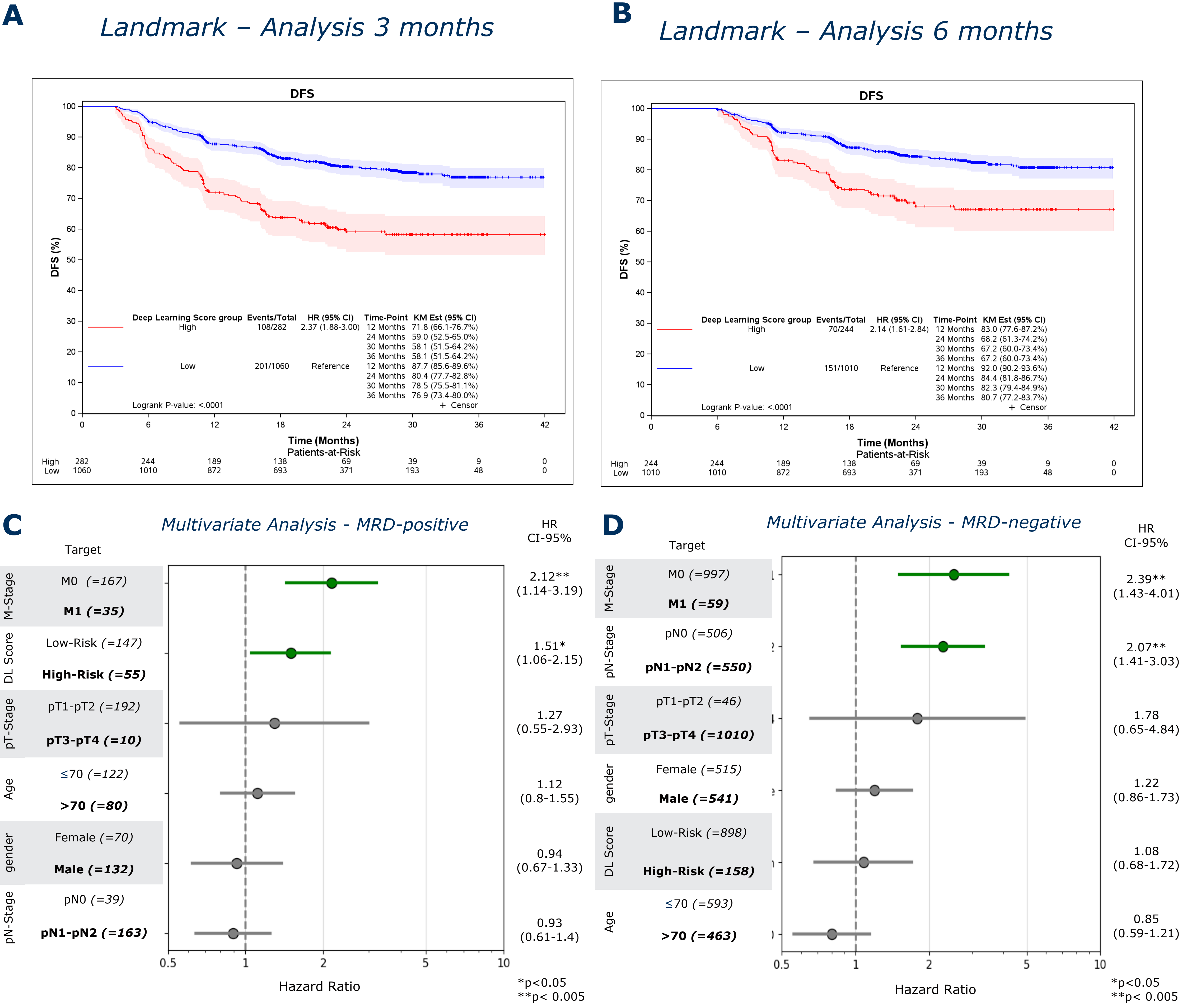

Supplementary Figure 2: MRD status is predictive of survival outcomes and Multivariate analysis for MRD-subgroups

Landmark Analysis for DFS stratified by DL high-risk and DL low-risk patients, excluding patients recurred within (A) three or (B) six months.

Forest plot showing multivariate cox regression analysis for (C) MRD-positive and (D) MRD-negative subgroup including the covariates gender, age, DL risk score, pathological Nodal Stage (pN-Stage), pathological Tumor Stage (pT-Stage), Metastasis stage (M-Stage) and their association with DFS. HR and 95% CI were calculated by the Cox proportional hazard model. *P*-value was calculated using the two-sided log-rank test (*p<0.05, ** p<0.001). Plot were generated using lifelines package in Python 3.11.5 DFS=disease-free survival, DL=Deep Learning, HR=Hazard ratio, CI=Confidence interval.

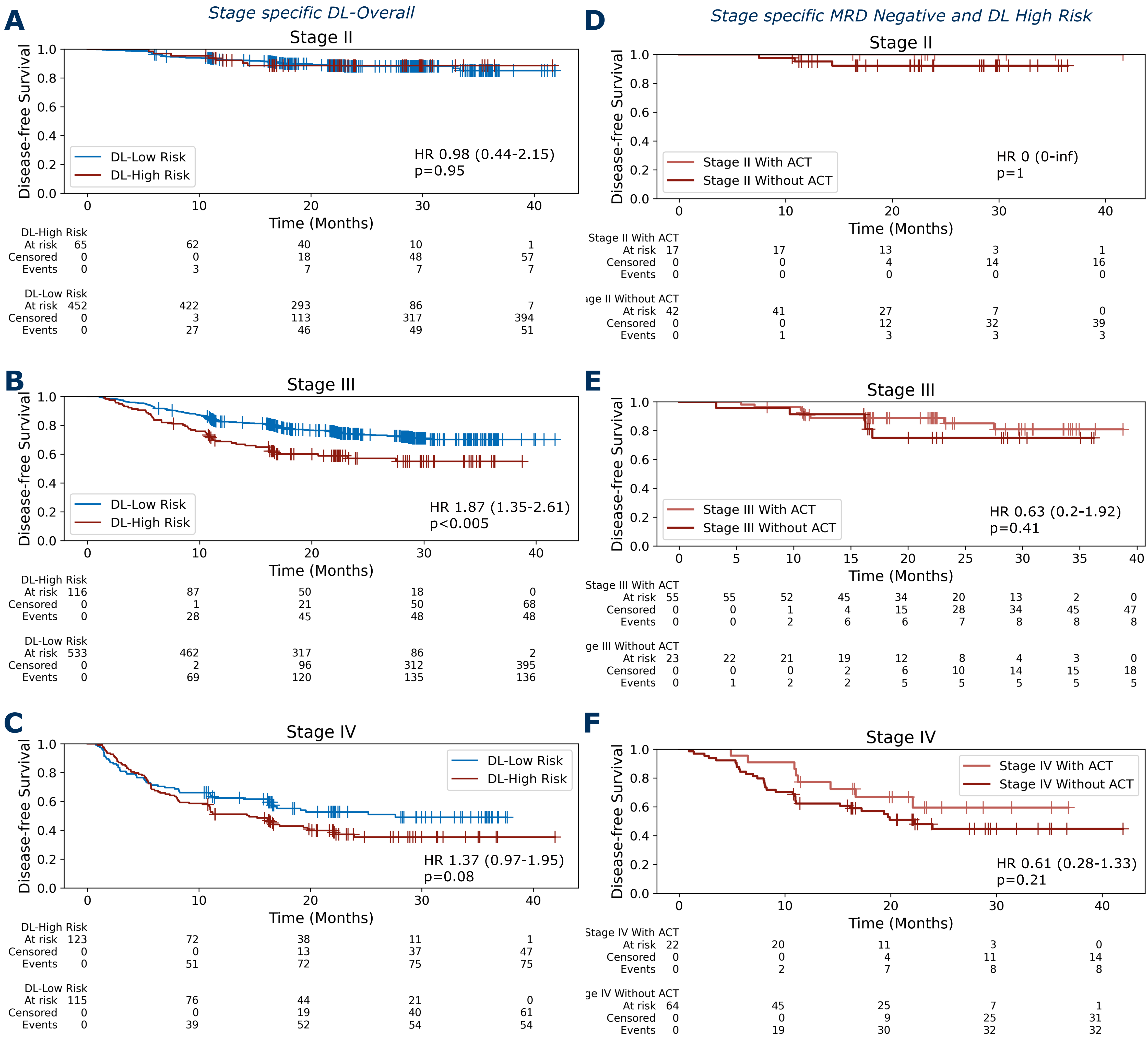

Supplementary Figure 3: Stage-Specific Prognostic Value of the DL Risk Score in CRC Patients

(A-C) Stage-Specific Kaplan-Meier curves for DFS (A-C) in the GALAXY cohort stratified by DL risk score and (D-F) in MRD-Negative and DL High-Risk patients stratified by ACT treatment. HR and 95% CI calculated by the Cox proportional hazard model. P-value was calculated using the two-sided log-rank test. Plots were generated using the lifelines package in Python 3.11.5 DFS=disease-free survival, DL=Deep Learning, ACT=adjuvant chemotherapy, MRD=molecular residual disease, HR=Hazard ratio, CI=Confidence interval.

Landmark – Analysis 3 months

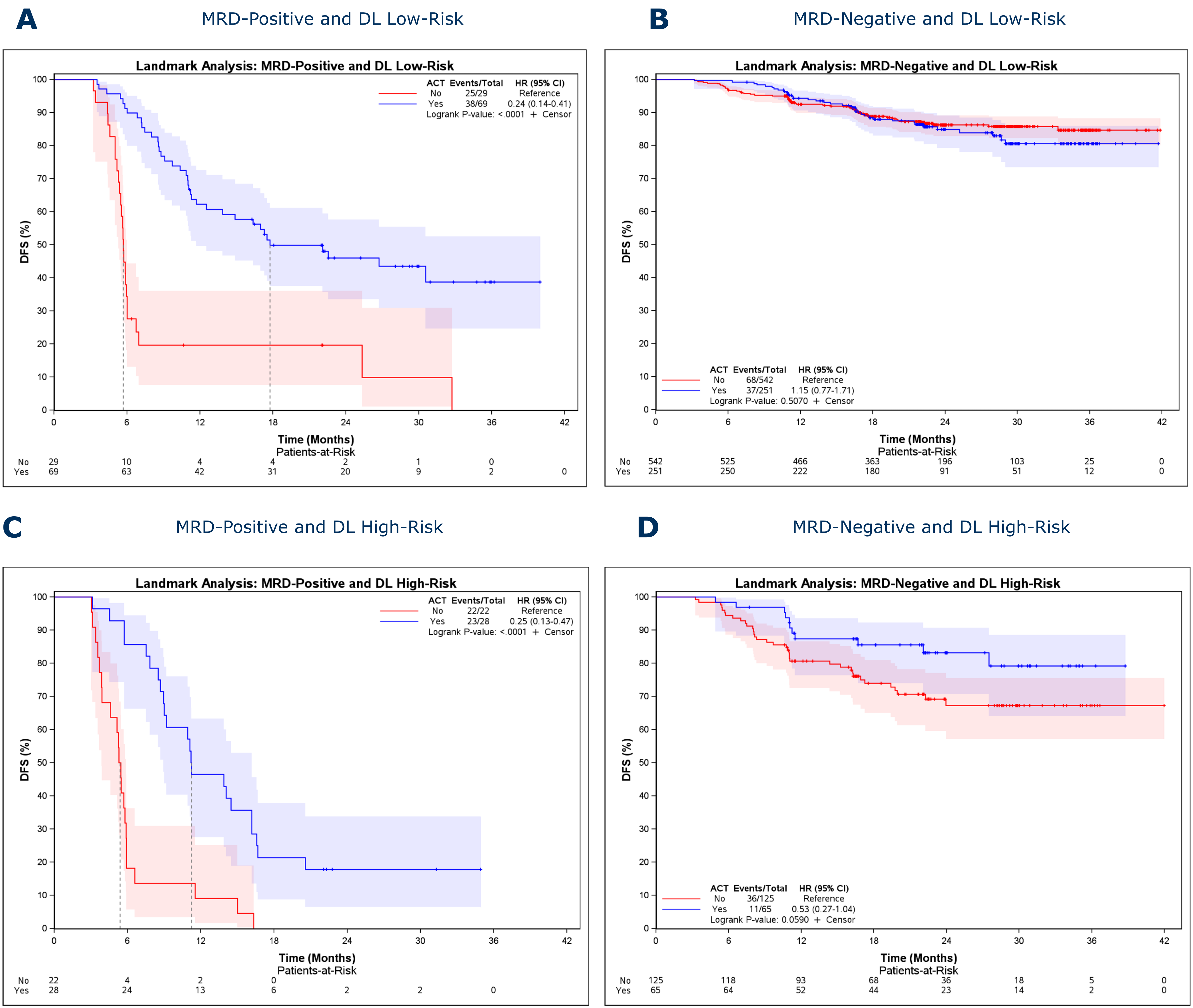

Supplementary Figure 4: Landmark Analysis of Disease-Free Survival Stratified by ACT Treatment

Landmark Analysis for DFS stratified by qith or without ACT treamtent excluding patients recurred within three months for in (A) MRD-positive and DL low-risk, (B) MRD-negative and DL low-risk, (C) MRD-positive and DL high-risk and (D) MRD-negative and DL high-risk subgroups. HR and 95% CI were calculated by the Cox proportional hazard model. *P*-value was calculated using the two-sided log-rank test. DFS=disease-free survival, DL=Deep Learning, ACT=adjuvant chemotherapy, MRD=molecular residual disease, HR=Hazard ratio, CI=Confidence interval

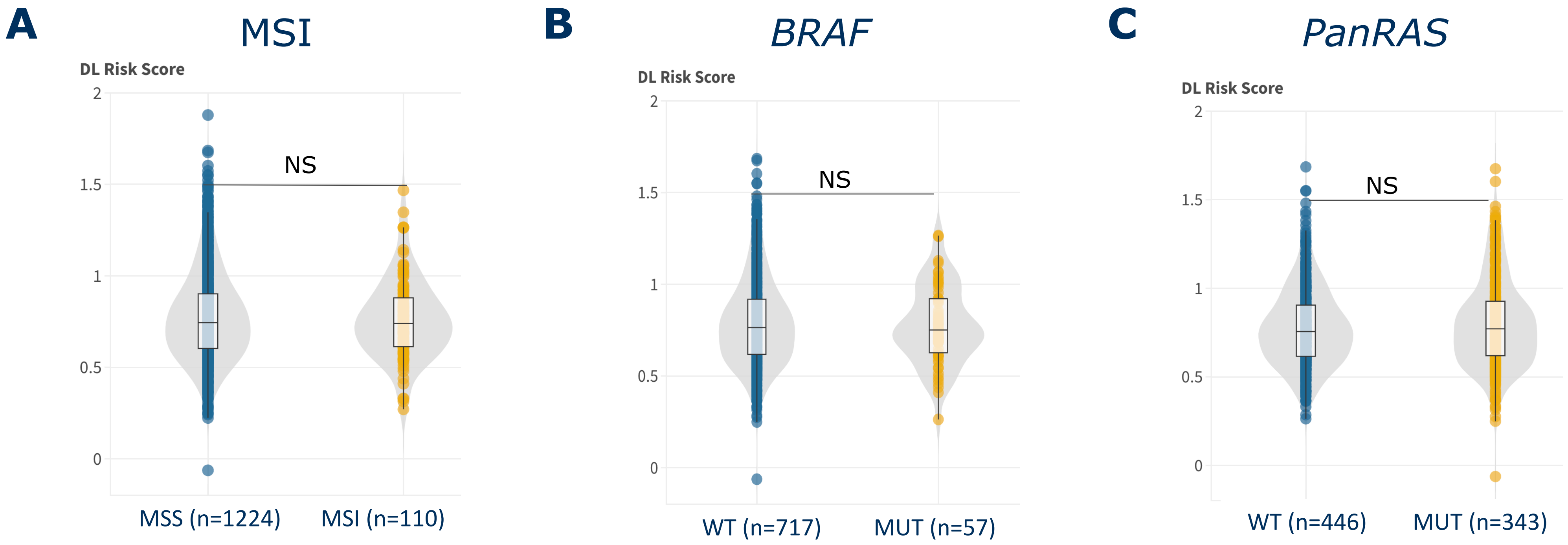

Supplementary Figure 5: DL Risk Score Distribution Independent of Key Molecular Subgroups

Box plot showing distribution of DL risk score among (A) MSI status (B) *BRAF-V600E* mutational status and (C) *PanRAS* mutational status. *P*-Value calculated using Kruskal-Wallis test. Box plots show the median (center line) and the interquartile range (box limits = 25th to 75th percentile). Outliers are not displayed. Figure was created using Flourish (https://flourish.studio/).

Supplementary Table 1: Patients characteristic of the DACHS and GALAXY cohort.
Moreover Patient characteristics of the GALAXY cohort stratified into DL high-risk and DL low-risk groups is included. P values were obtained using the Chi-Square two sided test. Statistical analysis was performed on R 4.4.4.
ECOG=Eastern Cooperativ Oncology Group, MSS=microsatellite stable, MSI=microsatellite instable, NA=Not available

Patient Characteristics	Category	DACHS		GALAXY		GALAXY		
		ALL (n=1,766)	n (%)	ALL (n=1404)	n (%)	DL-High-Risk (n=304), n (%)	DL-Low-Risk (n=1100), n (%)	*Chi squared and p values*
**Age**	≤70	962	54%	801	57%	180 (59.3%)	621 (56.5%)	*X² = 0.63 P-Val = 0.427*
	>70	804	46%	603	43%	124 (40.7%)	479 (43.5%)	
**Gender**	Female	753	43%	643	46%	118 (38.8%)	525 (47.7%)	*X² = 7.2649 P-Val = 0.007*
	Male	1013	57%	761	54%	186 (61.2%)	575 (52.3%)	
**ECOG performance**	0	-	-	1265	90%	277 (91.1%)	988 (89.8%)	*X² = 0.3174 P-Val = 0.5732*
	1	-	-	139	10%	27 (8.9%)	112 (10.2%)	
**Primary Site**	Left-sided colon	487	28%	681	49%	148 (48.7%)	533 (48.5%)	*X² = 1.1826 P-Val = 0.5536*
	Right-sided colon	634	36%	485	35%	99 (32.6%)	386 (35.1%)	
	Rectum	632	36%	238	17%	57 (18.8%)	181 (16.5%)	
	NA	13		0				
**pT-Stage**	T1-T2	106	6%	56	4%	7 (3.3%)	49 (4.7%)	*X² = 0.518 P-Val = 4.72E-01*
	T3-T4	1623	94%	1203	86%	206 (96.7%)	997 (95.3%)	
	NA	37		145	10%	91	54	
**pN-Stage**	N0	769	44%	545	39%	73 (34.3%)	472 (45.2%)	*X² = 8.116 P-Val = 4.00E-03*
	N1-2	963	56%	713	51%	140 (65.7%)	573 (54.8%)	
	NA	34		146	10%	91	55	
**AJCC Stage**	II	742	42%	517	37%	65 (21.4%)	452 (41.1%)	*X² = 157.0928 P-Val = 7.72E-35*
	III	723	41%	649	46%	116 (38.2%)	533 (48.5%)	
	IV	301	17%	238	17%	123 (40.5%)	115 (10.5%)	
***RAS* status**	*RAS* mutant	524	33%	343	24%	84 (46%)	259 (42.7%)	*X² = 0.547 P-Val = 0.46*
	RAS wild-type	1071	67%	446	32%	99 (54%)	347 (57.3%)	
	NA	171		615		121	494	

***BRAF* status**	*BRAF* mutant	122	8%	57	4%	13 (7.2%)	44 (7.4%)	*X² = 0* *P-Val = 1*
	BRAF wild-type	1475	92%	717	51%	168 (92.8%)	549 (92.6%)	
	NA	169		630	45%	123	507	
**MSI - status**	MSI	172	11%	110	8%	20 (7.2%)	90 (8.5%)	*X² = 0.376* *P-Val = 0.54*
	MSS	1388	89%	1224	87%	259 (92.8%)	965 (91.5%)	
	NA	206		70		25	45	
**MRD status**	NEGATIVE	-		1165	83%	223 (73.4%)	942 (85.6%)	*X² = 24.5702*
	POSITIVE	-		239	17%	81 (26.6%)	158 (14.4%)	*P-Val = 7.17E-07*
**Neoadjuvant Treatment**	No	1557	88%	1273	91%	238 (78.3%)	1035 (94.1%)	*X² = 68.44* *P-Val = 1.31E-16*
	Yes	205	12%	131	9%	66 (21.7%)	65 (5.9%)	
	NA	4		0				
**Adjuvant Chemotherapy**	No	828	47%	775	55%	167 (54.9%)	608 (55.3%)	*X² = 0.0016* *P-Val = 0.968*
	Yes	933	53%	629	45%	137 (45.1%)	492 (%)	
	NA	5		0				

Supplementary Table 2: Patients characteristic GALAXY cohort between patients who received ACT and those who did not

P-values were obtained using either the Chi-Square two sided test (¹) or the Wilcoxon rank sum test (²) for nonparametric values. ECOG=Eastern Cooperativ Oncology Group, MSS=microsatellite stable, MSI=microsatellite instable, NA=Not available

		Adjuvant Chemotherapy			
**Patient Characteristics**	**Category**	**No N=775 (%)**	**Yes N=629 (%)**	**Total N=1404 (%)**	***P-value***
**Deep Learning Score**	Low-Risk	608 (78.5%)	492 (78.2%)	1100 (78.3%)	0.91631
	High-Risk	167 (21.5%)	137 (21.8%)	304 (21.7%)	
**Location**	Ascending colon (A)	131 (16.9%)	113 (18.0%)	244 (17.4%)	0.62781
	Cecum (C)	56 (7.2%)	41 (6.5%)	97 (6.9%)	
	Descending colon (D)	40 (5.2%)	41 (6.5%)	81 (5.8%)	
	Lower rectum (Rb)	57 (7.4%)	37 (5.9%)	94 (6.7%)	
	Rectosigmoid (RS)	124 (16.0%)	110 (17.5%)	234 (16.7%)	
	Sigmoid colon (S)	208 (26.8%)	158 (25.1%)	366 (26.1%)	
	Transverse colon (T)	85 (11.0%)	59 (9.4%)	144 (10.3%)	
	Upper rectum (Ra)	74 (9.5%)	70 (11.1%)	144 (10.3%)	
**Primary Site**	Left-sided colon	372 (48.0%)	309 (49.1%)	681 (48.5%)	0.88431
	Rectum	131 (16.9%)	107 (17.0%)	238 (17.0%)	
	Right-sided colon	272 (35.1%)	213 (33.9%)	485 (34.5%)	
**Gender**	Female	360 (46.5%)	283 (45.0%)	643 (45.8%)	0.58521
	Male	415 (53.5%)	346 (55.0%)	761 (54.2%)	
**Age**	<=70	381 (48%)	420 (52%)	801 (57%)	<.0001[1]
	>70	394 (65%)	209 (35%)	603 (43%)	
**ECOG performance Status**	0	680 (87.7%)	585 (93.0%)	1265 (90.1%)	0.00101
	1	95 (12.3%)	44 (7.0%)	139 (9.9%)	
**AJCC Stage**	II	410 (52.9%)	107 (17.0%)	517 (36.8%)	<.0001[1]
	III	202 (26.1%)	447 (71.1%)	649 (46.2%)	

	IV	163 (21.0%)	75 (11.9%)	238 (17.0%)	
**pT-Stage**	T1	1 (0.2%)	6 (1.0%)	7 (0.6%)	<.0001[1]
	T2	21 (3.2%)	28 (4.6%)	49 (3.9%)	
	T3	508 (77.7%)	354 (58.5%)	862 (68.5%)	
	T4a	99 (15.1%)	184 (30.4%)	283 (22.5%)	
	T4b	25 (3.8%)	33 (5.5%)	58 (4.6%)	
	NA	121	24	145	
**pN-Stage**	N0	427 (65.3%)	118 (19.5%)	545 (43.3%)	<.0001[1]
	N1a	92 (14.1%)	146 (24.1%)	238 (18.9%)	
	N1b	77 (11.8%)	148 (24.5%)	225 (17.9%)	
	N1c	0 (0.0%)	4 (0.7%)	4 (0.3%)	
	N2a	25 (3.8%)	118 (19.5%)	143 (11.4%)	
	N2b	33 (5.0%)	70 (11.6%)	103 (8.2%)	
	NA	121	25	146	
**MSI - status**	MSI-HIGH	75 (10.2%)	35 (5.9%)	110 (8.2%)	0.00441
	MSS	662 (89.8%)	562 (94.1%)	1224 (91.8%)	
	NA	38	32	70	
***BRAF* status**	*BRAF* mutant	37 (9.5%)	20 (5.2%)	57 (7.4%)	0.02391
	BRAF wild-type	354 (90.5%)	363 (94.8%)	717 (92.6%)	
	NA	384	246	630	
***RAS* status**	*RAS* mutant	165 (41.4%)	178 (45.6%)	343 (43.5%)	0.22451
	RAS wild-type	234 (58.6%)	212 (54.4%)	446 (56.5%)	
	NA	376	239	615	
**MRD status**	NEGATIVE	677 (87.4%)	488 (77.6%)	1165 (83.0%)	<.0001[1]
	POSITIVE	98 (12.6%)	141 (22.4%)	239 (17.0%)	
**Adjuvant Chemotherapy**	No	775 (100.0%)	0 (0.0%)	775 (55.2%)	<.0001[1]
	Yes	0 (0.0%)	629 (100.0%)	629 (44.8%)	
[1]Chi-Square p-value; [2]Wilcoxon rank sum p-value;					
