# Supplementary material for: HIBRID: histology-based risk-stratification with deep learning and ctDNA in colorectal cancer
Source: Nat Commun. 2025 Aug 14;16:7561. doi: 10.1038/s41467-025-62910-8 (PMC12354865; doi:10.1038/s41467-025-62910-8)
Supplement: Supplementary file 3 — Reporting Summary [file 41467_2025_62910_MOESM3_ESM.pdf]

nature portfolio

Corresponding author(s): Jakob Nikolas Kather

Last updated by author(s): Jul 18, 2025

Reporting Summary

Nature Portfolio wishes to improve the reproducibility of the work that we publish. This form provides structure for consistency and transparency in reporting. For further information on Nature Portfolio policies, see our Editorial Policies and the Editorial Policy Checklist.

Statistics

For all statistical analyses, confirm that the following items are present in the figure legend, table legend, main text, or Methods section.

n/a	Confirmed	
☐	☒	The exact sample size (*n*) for each experimental group/condition, given as a discrete number and unit of measurement
☐	☒	A statement on whether measurements were taken from distinct samples or whether the same sample was measured repeatedly
☐	☒	The statistical test(s) used AND whether they are one- or two-sided<br>*Only common tests should be described solely by name; describe more complex techniques in the Methods section.*
☐	☒	A description of all covariates tested
☐	☒	A description of any assumptions or corrections, such as tests of normality and adjustment for multiple comparisons
☐	☒	A full description of the statistical parameters including central tendency (e.g. means) or other basic estimates (e.g. regression coefficient) AND variation (e.g. standard deviation) or associated estimates of uncertainty (e.g. confidence intervals)
☐	☒	For null hypothesis testing, the test statistic (e.g. *F*, *t*, *r*) with confidence intervals, effect sizes, degrees of freedom and *P* value noted<br>*Give P values as exact values whenever suitable.*
☒	☐	For Bayesian analysis, information on the choice of priors and Markov chain Monte Carlo settings
☒	☐	For hierarchical and complex designs, identification of the appropriate level for tests and full reporting of outcomes
☒	☐	Estimates of effect sizes (e.g. Cohen's *d*, Pearson's *r*), indicating how they were calculated

Our web collection on statistics for biologists contains articles on many of the points above.

Software and code

Policy information about availability of computer code

Data collection	No softwarte was used to collect the Data. Data was provided from external study Principal Investigators.
Data analysis	Survival Analysis was performed using Kapan-Meier analysis and log-rank test to compare DFS time between the groups. Additionally, multivariate analysis was conducted using Cox proportional hazard models, including the covariates: age, gender, pathological T-Stage (pT) and pathological N-Stage (pN)

For manuscripts utilizing custom algorithms or software that are central to the research but not yet described in published literature, software must be made available to editors and reviewers. We strongly encourage code deposition in a community repository (e.g. GitHub). See the Nature Portfolio guidelines for submitting code & software for further information.

Data

Policy information about availability of data

All manuscripts must include a data availability statement. This statement should provide the following information, where applicable:

- Accession codes, unique identifiers, or web links for publicly available datasets
- A description of any restrictions on data availability
- For clinical datasets or third party data, please ensure that the statement adheres to our policy

The respective study Principal Investigators provided the clinical and image data. Clinical, Sequencing and image data from the DACHS and GALAXY studies are available under restricted access due to ethical and legal constraints. For detailed data sharing policies, please refer to the original publications (https://

www.nature.com/articles/s41591-024-03254-6#data-availability; https://ascopubs.org/doi/10.1200/JCO.2011.35.9307?url_ver=Z39.88-2003&rfr_id=ori:rid:crossref.org&rfr_dat=cr_pub%20%200pubmed). 15,18,50 Requests for access to additional de-identified data can be submitted to the corresponding author and will be assessed by the steering committee within approximately 2–3 weeks. Data will be shared solely for the purpose of scientific validation and cannot be reused for other purposes. Source data are provided with this paper.

The code for the pretrained vision encoder UNI can be found under: https://github.com/mahmoodlab/uni. Our WSI preprocessing pipeline and DL model codes are publicly available at https://github.com/KatherLab/HIBRID under MIT license.

Research involving human participants, their data, or biological material

Policy information about studies with human participants or human data. See also policy information about sex, gender (identity/presentation), and sexual orientation and race, ethnicity and racism.

Reporting on sex and gender

In this study, we utilized two independent datasets from separate cohorts. The first cohort is the DACHS (Darmkrebs: Chancen der Verhütung durch Screening) cohort, which is a German population-based case-control study. Lifestyle and demographic data were obtained through in-person interviews with the participants. All potentially eligible patients were recruited either directly by their treating physicians or via postal invitations. In the DACHS cohort, the distribution of participants is 43% female and 57% male.
The second cohort includes data from the GALAXY trial (Clinical trial information: UMIN000039205), which enrolled patients starting in 2020. Participants self-reported their biological sex on the requisition form at the time of enrollment. In this cohort, 46% of the participants are female, and 54% are male. Pregnant women were not included in both trials.
Sex and gender were considered in the study design, and gender was included in the multivariate analysis. The distribution of sex and gender data is available in the patient characteristics table. Consent for data sharing was obtained. Patient sex was self-reported by study participants. No compensation was provided to participants.

Reporting on race, ethnicity, or other socially relevant groupings

No specific socially constructed variables, such as race or ethnicity, were used in this study. The two cohorts included in the analysis were recruited from distinct geographic regions, with the DACHS cohort enrolled in Germany and the GALAXY cohort enrolled in Japan. Consequently, the DACHS cohort predominantly represents a Western population, while the GALAXY cohort primarily reflects an Asian population. Participants in the DACHS cohort were eligible for inclusion if they were residents of Germany and German-speaking, while no such eligibility criteria were specified for the GALAXY trial.

All data regarding demographic variables were self-reported by the participants in both cohorts. These variables were not used as proxies for other socially constructed factors

Population characteristics

Key population characteristics considered in this study include age, sex, and clinical treatment information. Age at enrollment was recorded for all participants in both cohorts. In the DACHS cohort, additional informations such as lifestyle, family history of cancer, and previous medical conditions were collected through in-person interviews. Current and past treatments were documented for both cohorts. Pathological stage was obtained from histopathological examination for both cohorts.
For the GALAXY cohort, clinical data such as treatment categories, disease stage, and response to treatment were collected based on the clinical trial protocol. RAS and BRAFV600E mutational status and microsatellite instability (MSI) for the GALAXY cohort were measured using the MEBGEN RASKET-B KIT (Medical & Biological Laboratories) and a polymerase chain reaction-based MSI test (SRL) at a central laboratory.
In the DACHS cohort, microsatellite instability (MSI-high) was determined using a mononucleotide marker panel (BAT25, BAT26, CAT25) in sections of the tumor block. Additionally, KRAS/ BRAF mutations were screened using a single-stranded conformational polymorphism technique (SSCP) on DNA extracted from the same tumor tissue samples.
These covariates were incorporated in the statistical models to adjust for potential confounders and to better understand the relationships between clinical variables and outcomes. Detailed patient characteristics, including distributions of demographic and clinical variables, are provided in the manuscript for both cohorts.

Recruitment

The GALAXY cohort is a prospective observational arm of the broader international CIRCULATE study, which aims to evaluate the clinical utility of circulating tumor DNA (ctDNA) analysis in patients with resectable colorectal cancer (CRC). This study is part of the CIRCULATE-Japan project, a large-scale platform that enrolled patients across more than 92 institutions. Detailed information about the project is available here: (https://www.nature.com/articles/s41591-022-02115-4).

The DACHS (Darmkrebs: Chancen der Verhütung durch Screening) study is a German population-based, case-control study initiated in 2003 in the Rhine-Neckar-Odenwald region in southwest Germany. Participants were randomly selected based on their age, sex, and county of residence from population registries. The recruitment process involved contacting potentially eligible patients through their treating physicians or via mail, with an estimated inclusion rate of over 50% of eligible patients. Recruitment was conducted in collaboration with all regional hospitals to ensure comprehensive participation and coverage of the local population.

Ethics oversight

The experiments in this study were carried out according to the Declaration of Helsinki and the International Ethical Guidelines for Biomedical Research Involving Human Subjects by the Council for International Organizations of Medical Sciences (CIOMS). The present study also adheres to the “Transparent reporting of a multivariable prediction model for individual prognosis or diagnosis” (TRIPOD) statement.20. The Ethics Board at the Medical Faculty of Technical University Dresden (BO-EK-444102022) and Institutional Review Board of the National Cancer Center Japan (2023-207) approved of the overall analysis in this study. The patient sample collection in each cohort was separately approved by the respective institutional ethics board. All patients provided written informed consent before participation in the study.

Note that full information on the approval of the study protocol must also be provided in the manuscript.

Field-specific reporting

Please select the one below that is the best fit for your research. If you are not sure, read the appropriate sections before making your selection.

☒ Life sciences ☐ Behavioural & social sciences ☐ Ecological, evolutionary & environmental sciences

For a reference copy of the document with all sections, see nature.com/documents/nr-reporting-summary-flat.pdf

Life sciences study design

All studies must disclose on these points even when the disclosure is negative.

Sample size	A formal sample size calculation was not performed for this study. Instead, all available data provided by collaborators were utilized to maximize the robustness and generalizability of the results. The rationale for using the entire dataset is based on established knowledge that for deep learning models, larger sample sizes contribute to improved performance and model stability. The dataset size was therefore considered sufficient for our analysis as it captures a diverse range of patient characteristics, enhancing the statistical power and validity of the findings.
Data exclusions	For the DACHS cohort, patients with stage I colorectal cancer were excluded to ensure comparability with the GALAXY cohort, which only included patients with stage II to resectable stage IV disease. Additionally, patients with missing imaging data or incomplete clinical information were excluded from the analysis. A detailed CONSORT diagram illustrating the exclusions and final sample size is provided in Supplementary Figure 2.
Replication	The exact study can only be fully replicated using the patient data, which is not publicly available at this time due to data privacy and confidentiality agreements. However the deep learning algorithms developed and applied in this study have been made fully open source. This allows other researchers to reuse, adapt, and build upon the models using their own datasets. Detailed descriptions of the model architecture, training process, and parameters are provided in the manuscript and accompanying code repository, enabling others to replicate and validate the findings in similar contexts.
Randomization	Randomization was not applicable in this study as two independent datasets were used: the DACHS cohort, a population-based case-control study, and the GALAXY cohort, a prospective observational study. The DACHS cohort was used as the training cohort due to the lack of ctDNA values, which were necessary for the primary analysis. The GALAXY cohort served as an independent external validation cohort to evaluate the generalizability and robustness of the developed models. To control for potential biases and ensure comparability, relevant covariates such as age, sex, and clinical stage were accounted for in the statistical analyses.
Blinding	Blinding was not relevant to this study because the analysis was performed on pre-existing retrospective data. No interventions or treatments were assigned by the researchers, and no subjective measurements were made that could introduce bias.

Reporting for specific materials, systems and methods

We require information from authors about some types of materials, experimental systems and methods used in many studies. Here, indicate whether each material, system or method listed is relevant to your study. If you are not sure if a list item applies to your research, read the appropriate section before selecting a response.

Materials & experimental systems

n/a	Involved in the study	
☒	☐	Antibodies
☒	☐	Eukaryotic cell lines
☒	☐	Palaeontology and archaeology
☒	☐	Animals and other organisms
☐	☒	Clinical data
☒	☐	Dual use research of concern
☒	☐	Plants

Methods

n/a	Involved in the study	
☒	☐	ChIP-seq
☒	☐	Flow cytometry
☒	☐	MRI-based neuroimaging

Clinical data

Policy information about clinical studies

All manuscripts should comply with the ICMJE [guidelines for publication of clinical research](guidelines) and a completed CONSORT checklist must be included with all submissions.

Clinical trial registration	*Provide the trial registration number from ClinicalTrials.gov or an equivalent agency.*
Study protocol	*Note where the full trial protocol can be accessed OR if not available, explain why.*
Data collection	*Describe the settings and locales of data collection, noting the time periods of recruitment and data collection.*

Outcomes

Describe how you pre-defined primary and secondary outcome measures and how you assessed these measures.

Plants

Seed stocks

Report on the source of all seed stocks or other plant material used. If applicable, state the seed stock centre and catalogue number. If plant specimens were collected from the field, describe the collection location, date and sampling procedures.

Novel plant genotypes

Describe the methods by which all novel plant genotypes were produced. This includes those generated by transgenic approaches, gene editing, chemical/radiation-based mutagenesis and hybridization. For transgenic lines, describe the transformation method, the number of independent lines analyzed and the generation upon which experiments were performed. For gene-edited lines, describe the editor used, the endogenous sequence targeted for editing, the targeting guide RNA sequence (if applicable) and how the editor was applied.

Authentication

Describe any authentication procedures for each seed stock used or novel genotype generated. Describe any experiments used to assess the effect of a mutation and, where applicable, how potential secondary effects (e.g. second site T-DNA insertions, mosiacism, off-target gene editing) were examined.
